# Molybdenum Reduction to Molybdenum Blue in *Serratia* sp. Strain DRY5 Is Catalyzed by a Novel Molybdenum-Reducing Enzyme

**DOI:** 10.1155/2014/853084

**Published:** 2014-03-03

**Authors:** M. Y. Shukor, M. I. E. Halmi, M. F. A. Rahman, N. A. Shamaan, M. A. Syed

**Affiliations:** ^1^Department of Biochemistry, Faculty of Biotechnology and Biomolecular Sciences, Universiti Putra Malaysia (UPM), 43400 Serdang, Selangor, Malaysia; ^2^Faculty of Medicine and Health Sciences, Universiti Sains Islam Malaysia, 13th Floor, Menara B, Persiaran MPAJ, Jalan Pandan Utama, Pandan Indah, 55100 Kuala Lumpur, Malaysia

## Abstract

The first purification of the Mo-reducing enzyme from *Serratia* sp. strain DRY5 that is responsible for molybdenum reduction to molybdenum blue in the bacterium is reported. The monomeric enzyme has an apparent molecular weight of 105 kDalton. The isoelectric point of this enzyme was 7.55. The enzyme has an optimum pH of 6.0 and maximum activity between 25 and 35°C. The Mo-reducing enzyme was extremely sensitive to temperatures above 50°C (between 54 and 70°C). A plot of initial rates against substrate concentrations at 15 mM 12-MP registered a *V*_max_ for NADH at 12.0 nmole Mo blue/min/mg protein. The apparent *K*_*m*_ for NADH was 0.79 mM. At 5 mM NADH, the apparent *V*_max_ and apparent *K*_*m*_ values for 12-MP of 12.05 nmole/min/mg protein and 3.87 mM, respectively, were obtained. The catalytic efficiency (*k*_cat_/*K*_*m*_) of the Mo-reducing enzyme was 5.47 M^−1^ s^−1^. The purification of this enzyme could probably help to solve the phenomenon of molybdenum reduction to molybdenum blue first reported in 1896 and would be useful for the understanding of the underlying mechanism in molybdenum bioremediation involving bioreduction.

## 1. Introduction

Microbes are at the forefront of heavy metals bioremediation due to their amazing ability to resist the inhibitory effects of heavy metals through a variety of mechanisms including bioprecipitation, extra- and intracellular sequestration, biosorption, bioreduction, transport mechanisms, and/or chelation [1].

One emerging global metal pollutant is molybdenum [2]. Microbial molybdenum reduction to molybdenum blue, a potential bioremediation tool, is a phenomenon that has been reported for over a century. According to Levine [3], microbial molybdate reduction to molybdenum blue was first mentioned in 1896 in *E. coli* [4]. Detailed studies on this phenomenon was only initiated in 1985 by Campbell et al. in *E. coli* K12 [5]. The reduction of molybdate into molybdenum blue by a chemolitotroph, *Thiobacillus ferrooxidans* (now *Acidithiobacillus ferrooxidans*) strain AP19-3, was reported by Sugio et al. [6] without citing the works carried out by Campbell et al. [5]. This indicates the rarity of publications over this phenomenon. The first local bacterium reported with molybdenum-reducing ability is *Enterobacter cloacae* strain 48 (EC 48) [7]. A purification of the molybdenum-reducing enzyme was attempted by Ariff et al. [8] but the activity is lost beyond the ammonium sulphate fraction. It was later discovered that the molybdenum blue produced from EC 48 and various other molybdenum-reducing bacteria exhibited a unique absorption spectra very similar to a reduced phosphomolybdate spectrum, thereby indicating that the intermediate species, phosphomolybdate, is involved [9–16]. Yong et al. [17] demonstrates that molybdenum reduction to molybdenum blue in *Thiobacillus ferrooxidans* is likely due to chemical action of ferrous irons supplemented in the medium. Up to this point, no method has yet been developed for distinguishing between genuine enzymatic and chemical reductions that has often plagued microbiological metal-reduction phenomenon [18]. Fortunately, using a modified dialysis tubing method, the reduction of molybdenum to molybdenum blue in EC 48 and other molybdenum-reducing heterotrophic bacteria is demonstrated to be enzymatically linked [9–16, 18]. A novel enzyme assay using phosphomolybdate instead of molybdate was developed and a partial purification of the enzyme using ammonium sulphate precipitation, ion exchange, and gel filtration was attempted [19]. More recently, a better assay was constructed using laboratory-prepared phosphomolybdate [20] similar to the assay developed by Glenn and Crane [21]. In this work, the purification and characterisation of the Mo-reducing enzyme from *Serratia* sp. strain Dr.Y5 are here presented for the first time. It is hoped that this will increase the understanding of the outlying reduction mechanism of molybdenum to the nontoxic molybdenum blue in microbes that was reported as early as about 120 years ago.

## 2. Methods

### 2.1. Chemicals

All chemicals used were of analytical grade. Preparation of buffers was carried out at the appropriate temperatures by mixing the appropriate dibasic and basic salts.

### 2.2. Growth and Maintenance of Molybdate-Reducing Bacterium

The bacterium was isolated from the city of Taiping, Perak, Malaysia [14]. The bacterium was maintained on a solid agar of low phosphate (2.9 mM phosphate) media (pH 7.0) containing (w/v%) sucrose (1%), MgSO_4_·7H_2_O (0.05%), (NH_4_)_2_SO_4_ (0.3%), yeast extract (0.05%), NaCl (0.5%), Na_2_MoO_4_·2H_2_O (0.726%), and Na_2_HPO_4_ (0.073%). Sucrose was autoclaved separately. Growth in liquid media used 100 mM phosphate instead.

### 2.3. Preparation of Crude Mo-Reducing Enzyme Fraction

The following experiment was carried out at 4°C unless stated otherwise. Cells of *Serratia* sp. strain DRY5 were harvested from a 5 L media (100 mM phosphate media) through centrifugation at 10 000 g for 10 minutes after growth at 30°C for 24 hours on an orbital shaker at 100 rpm. Cells were resuspended with deionised water and centrifuged at 15 000 g for 10 minutes. This process was repeated twice. The pellet was reconstituted with 50 mL of 50 mM Tris-HCl buffer pH 7.5 containing 0.5 mM dithiothreitol and 0.1 mM PMSF (phenyl-methane-sulfonyl-fluoride). Cells were sonicated (Branson) on ice and then centrifuged at 15 000 g for 20 minutes. Sonication was considered complete when little pellet was formed [19]. The supernatant is crude *Serratia* sp. strain DRY5 cell fraction. The crude fraction was subjected to ultracentrifugation for 2 hours at 105 000 g. The supernatant contains high Mo-reducing activity.

### 2.4. Enzyme Assay

The reaction mixture, 1 mL, contained 3 mM of 12-MP (electron acceptor substrate) in 50 mM citrate phosphate buffer pH 5.0 at room temperature and 100 *μ*L of NADH at the final concentration of 3 mM. Fifty microlitres of enzyme fraction containing about 1 mg protein was added to start the reaction. The absorbance increase in one minute was read at 865 nm. One unit of Mo-reducing enzyme activity is defined as the amount of enzyme that produces 1 nmole molybdenum blue measured as equivalent to ascorbate-reduced 12-MP in one minute at room temperature. The molar absorptivity or extinction coefficient at 865 nm for molybdenum blue using 12-MP as a standard is 16.7 mM ^−1^·cm^−1^. An increase in 1.00 unit absorbance per minute at OD 865 nm of per mg protein would yield 60 units of enzyme activity or 60 nmole of 12-MP in a 1 mL assay mixture [20].

### 2.5. Ion-Exchange Chromatography Using Mono-Q Strong-Anion Exchanger

Although ammonium sulphate gave good purification and yield in the purification attempt of Mo-reducing enzyme from EC 48 [19], preliminary results show that it gave no advantage in purification fold (3-fold) and also gave poor recovery (<50%). All experiments were carried out at 4°C unless stated otherwise. Thus, crude fraction (cytoplasmic fraction after ultracentrifuge) was subjected straight to ion exchange. The crude fraction was subjected to the strong-anion exchange matrix Mono Q (Amersham Pharmacia). The Mono-Q 5/50 GL Tricorn column (maximum tolerable pressure of 50 Bar) was connected to a modified Agilent 1100 Series high performance liquid chromatography (HPLC) unit. A makeshift cooling system consisting of a 4°C chilled circulating water through silicon tubing system was developed to cool the column. The original HPLC loop and tubings were replaced with polyetheretherketone (PEEK) tubings. The column was first washed with 5 mLs of 1 M NaCl in buffer A followed by washing with 200 mL of buffer A until the eluant pH is 7.5. About 40 milligrams of the crude enzyme in 2 mLs of volume was injected into Rheodyne sample injector to load the Mono-Q column at a flow rate of 1 mL per minute and then washed with the same buffer until the signal for protein content, measurable at 280 nm, became undetected. The washed eluant was collected using a fraction collector at 1 mL per tube and assayed for enzyme activity. Enzyme was eluted from the column with a linear gradient of 0–0.5 M KCl in buffer A at the flow rate of 1 mL min^−1^. The protein elution profile was monitored at 280 nm.

The fractions showing enzyme activity were pooled and assayed for protein and enzyme activity. The pooled enzyme was dialyzed against 5 L of 10 mM Tris. Cl pH 7.5 containing 0.5 mM dithiothreitol for 5 hours. The dialysed fraction was centrifuged at 15 000 g to remove precipitated fraction and the supernatant applied again to Mono Q and purification of the enzyme was carried out as before. Protein was quantified according to the method of Bradford [22] using BSA as the standard. The dialyzed enzyme was then concentrated using a cellulose triacetate filter membrane with a molecular weight cut-off point of 10 kDa in an Amicon ultrafiltration cell at 4°C to a final volume of 0.5 mL. One hundred microlitres of sample was applied into Zorbax GFC-250 column (250 × 9.4 mm) and eluted using buffer A containing 0.2 M KCL at a flow rate of 0.5 mL min^−1^. The purified fraction was used for kinetic studies.

### 2.6. Determination of Kinetic Parameter

Michaelis menten kinetics constants were determined using GraphPad prism nonlinear regression analysis available from http://www.graphpad.com/.

### 2.7. Molecular Mass Determination

Estimation of the native molecular weight of the Mo-reducing enzyme was carried out using gel filtration on Zorbax GF-250 precalibrated with gel filtration molecular weight markers (Bio Rad) and the subunit Mr was determined using SDS–PAGE. Broad range protein standard marker (BioRAd) (myosin (200 kD), *β*-galactosidase (116.25 kD), bovine serum albumin (66.2 kD), phosphorylase b (97.4 kD), ovalbumin (45 kD), soybean trypsin inhibitor (21.5 kD), carbonic anhydrase (31 kD), aprotinin (6.5 kD), and lysozyme (14.4 kD)) were used to determine the molecular weight of the protein. Proteins in the gels were detected with silver staining [23].

### 2.8. Estimation of the Isoelectric Point (pI)

The pI of the enzyme was estimated by chromatofocusing on Mono P 5/200 GL Tricorn column (Pharmacia Biotech) anion exchange column attached to an Agilent 1100 series. This was equilibrated with 25 mM diethanolamine pH 9.5 containing 0.5 mM dithiothreitol until the eluant has a pH of 9.5. The column was eluted (pregradient) with 9 mL of an isocratic gradient of 10% (v/v) Polybuffer 96 adjusted to pH 6.0 with HCl before sample was injected into the column and eluted using the same buffer [24].

## 3. Results and Discussion

### 3.1. Purification of Mo-Reducing Enzyme

**Figure 1:**
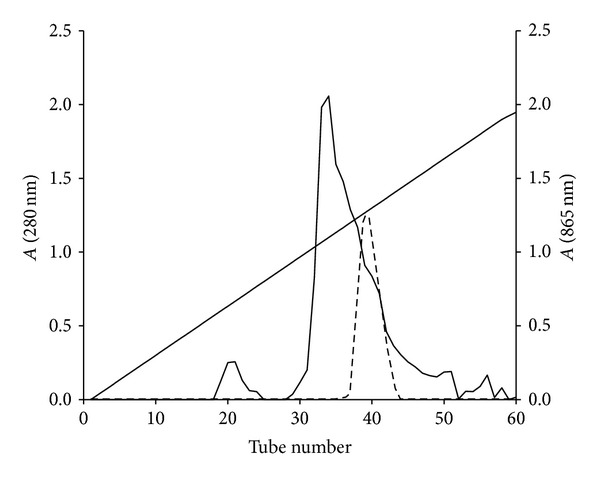
Ion exchange on Mono Q. Elution profile of protein (- - - -) and enzyme activity (**—**). Diagonal line represents salt (NaCl) gradient (0 to 0.5 M).

**Figure 2:**
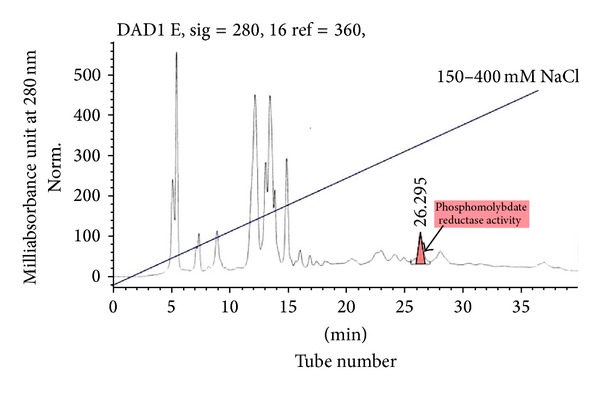
Second ion exchange of Mo-reducing enzyme on Mono Q on an Agilent 1100 series. Shaded region indicates Mo-reducing enzyme activity.

**Figure 3:**
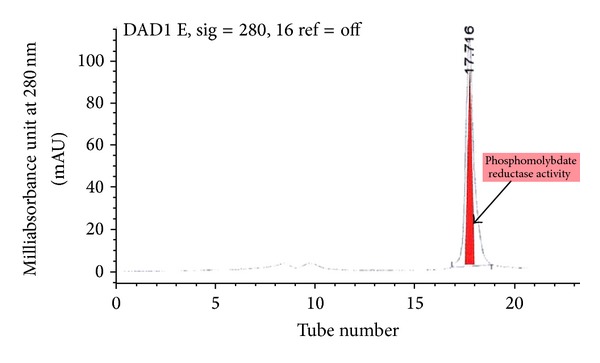
Gel filtration on Zorbax GFC-250. Shaded region indicates Mo-reducing enzyme activity. Each tube number or fraction represents 0.5 mL.

**Figure 4:**
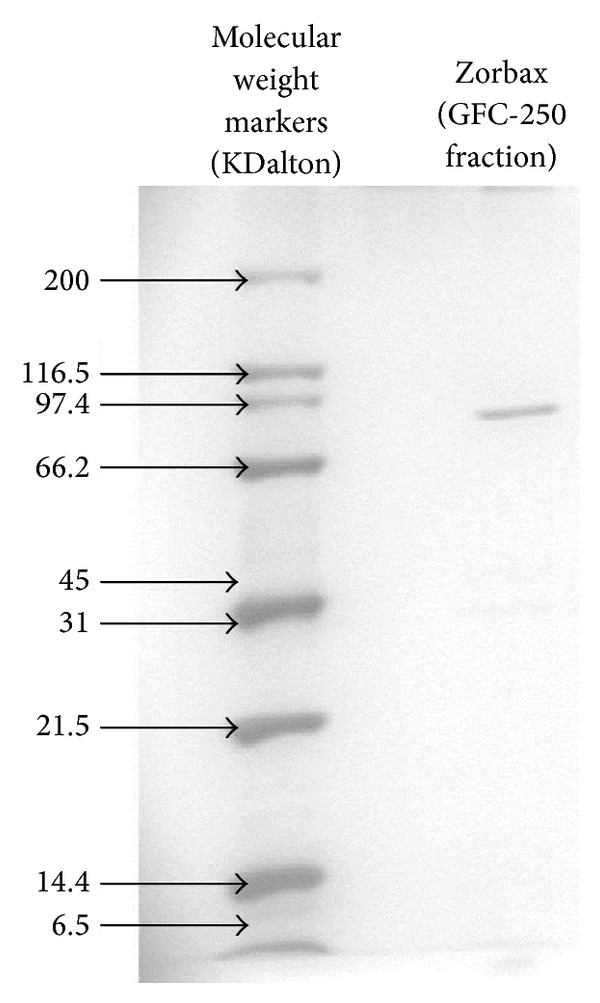
SDS-PAGE electrophoretogram of purified Mo-reducing enzyme stained by silver staining method.

The fraction containing enzyme activity elutes at tube number 19 to 20 at about 330 mM NaCl (Figure 1). This fraction was pooled, dialysed, and rechromatographed again on Mono Q. The results show more improvement in terms of the separation of Mo-reducing enzyme from the enzyme eluting as a single peak at tube number 26 at 300 mM NaCl (Figure 2). A single peak was also seen on gel filtration when the fraction with Mo-reducing enzyme activity from the second ion exchange was applied to Zorbax GF-250 (Figure 3). An 8.1-fold purification was achieved after gel filtration. A 40-fold partial purification was achieved after gel filtration on Sephadex G-200 in EC 48. The apparent molecular weight for the Mo-reducing enzyme as estimated from gel filtration was 105 KDalton. Native-PAGE analysis revealed that the presence of only a single band near (data not shown) while denaturing SDS-PAGE shows a single band at 100 KDa (Figure 4). Together with the gel filtration results, it can be concluded that the Mo-reducing enzyme was monomeric.  Table 1 shows that ion exchange chromatography removes much of the enzyme activity. This results in a markedly reduced yield. The isoelectric point of this enzyme was determined to be 7.55 using Mono P chromatofocusing. Several attempts to purify the Mo-reducing enzyme from EC 48 have resulted in failure due to the problematic assay system [7, 8] and to the problematic ion exchange step [19].

The ion exchange stage removes much of enzyme activity but the method was vital in the purification strategy. The same effect was also observed during the purification of the enzyme from EC 48. Probably, a soluble coenzyme was removed during adsorption to the exchanger. In the foreseeable future, other chromatographic techniques and studies on the effects of possible cofactors or coenzymes will be carried out to address this issue. A partial purification of the enzyme from EC 48 showed three protein bands with the molecular weights of 80, 90 and 100 kDa [19]. The purification step using Mono P showed no improvement in the purification fold and the yield was very poor. Probably, the enzyme was no longer stable after gel filtration.

Phosphomolybdate reduction to molybdenum blue by enzymatic reaction has previously been reported, catalysed by xanthine and aldehyde oxidase [21], although preliminary results using xanthine and formaldehyde (or acetaldehyde as an electron donor substrates) did not yield Mo-blue in this bacterium, indicating that a different enzyme is responsible. This is the first report on the purification of an enzyme responsible for the physiological molybdate reduction seen in microbes.

To date, the enzyme responsible for the Mo-reducing activity in EC 48 or any other bacterium has never been successfully purified to homogeneity.

The only known enzyme which could reduce molybdate is molybdate reductase [25]. The enzyme catalyses the reduction of Mo(6+) to the (4+) oxidation state before its integration with the sulphur atoms of a pterin derivative named molybdopterin, cofactor of molybdoenzymes. During the enzyme-catalysed reaction, the oxidation state of molybdenum changes so that molybdenum is involved in the electron-transfer pathway. Molybdenum cofactor-containing enzymes catalyse the transfer of an oxygen atom, ultimately derived from or incorporated into water, to or from a substrate in a two-electron redox reaction [26]. The reduction of molybdate to the 4+ oxidation state is not accompanied by a change in colour. The oxidation state of molybdenum blue is, however, quite complex. Electron spin resonance (esr) work showed that the reducing agent dithionite donates two electrons to a heteropolymolybdate, PMo_12_O_40_^3−^ (12-molybdophosphate), producing Mo-blue. The introduced electrons were found to be uniformly dispersed over the whole polymetallate sphere by a process involving thermal activation hopping. The electrons in the two-electron reduced forms were very mobile as shown by ^17^O nuclear magnetic resonance (nmr) spectroscopy. This results in the averaging of the valence of all the twelve molybdenum atoms [27]. This explains the resultant mixed valence (between 5+ and 6+) properties of Mo-blue [28].

### 3.2. Optimum pH

**Figure 5:**
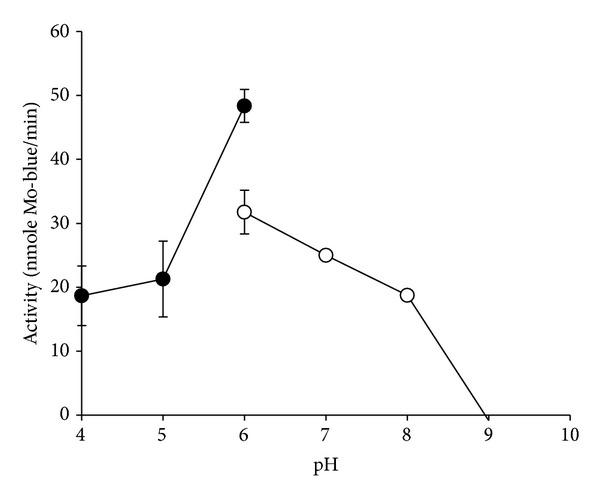
Effect of pH on enzyme activity using an overlapping buffer system consisting of citrate (●) and phosphate (◯) buffers. Error bars are mean ± standard deviation of triplicates.

The activity of the purified enzyme was measured at various pH values ranging between 4.0 and 9.0. As shown in Figure 5, maximum activity was obtained at pH 6.0. In contrast, Mo-reducing activity in EC 48 occurs optimally at pH 5.0 [19]. It has been reported that formation of 12-MP and heteropolymolybdates, in general, requires an acidic environment and that 12-MP is unstable at neutral pH [28]. This could explain the low optimum pH for the reaction of the enzyme on 12-MP for both bacteria, since the substrate is not stable at neutral and higher pH. The lower activity exhibited by phosphate buffer is possibly due to the effect of phosphate on phosphomolybdate instability [18].

### 3.3. Optimum Temperature

**Figure 6:**
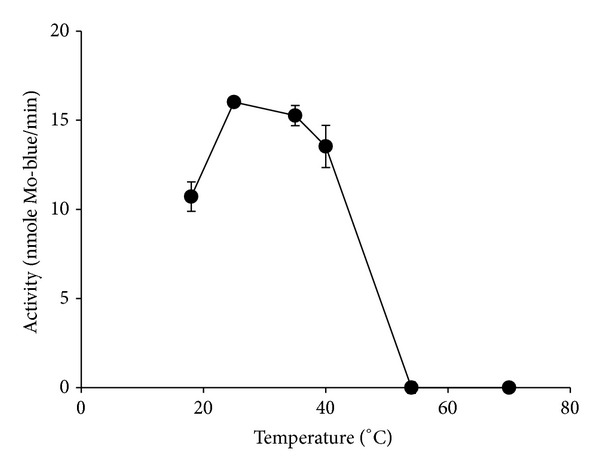
Effect of temperature on enzyme activity. Error bars are mean ± standard deviation of triplicates.

To determine the optimum temperature for enzyme activity, reactions were performed at various temperatures (20–70°C) at pH 6.0 for 1 h. The Mo-reducing enzyme showed maximum activity in between 25 and 35°C (Figure 6) similar to the optimum temperature range reported for EC 48 (28–33°C) [19]. The activity drastically dropped at higher temperatures and no activity was detected higher than 50°C. The profile of optimum temperature fits well for most mesophilic bacterium with activity ranges from 20 to 40°C.

### 3.4. Temperature Stability Studies

**Figure 7:**
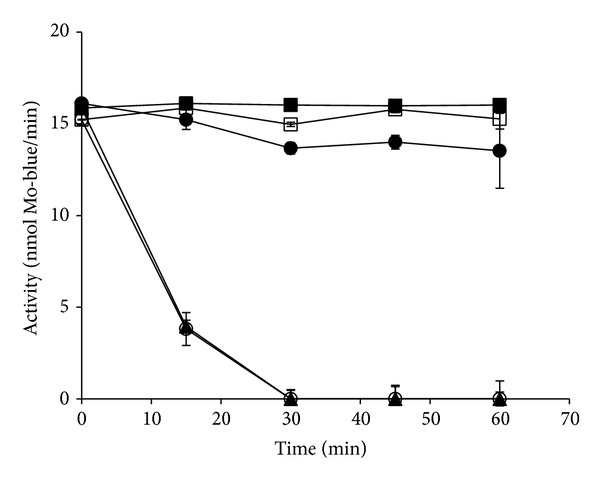
The effects of preincubation temperatures of 25°C (■), 34°C (□), 40°C (●), 54°C (▲), and 70°C (◯) on the stability of the enzyme. Error bars are mean ± standard deviation of triplicates.

Mo-reducing enzyme was extremely sensitive to temperatures above 50°C (54 and 70°C) as evident from Figure 7 with total loss of activity occurring after 30 minutes of preincubation. Other preincubation temperatures (25 to 40°C) caused 80% loss of enzyme activity after 15 hours of incubation (Data not shown). The instability of enzymes at high temperature is caused by several factors including tertiary and quaternary protein denaturation through thermal vibration leading to loss of cofactors, contaminating protease (from handling) with accelerated activity at higher temperatures, and accelerated oxidation of sulfhydryl groups at higher temperatures to name a few [19].

### 3.5. pH Stability Studies

**Figure 8:**
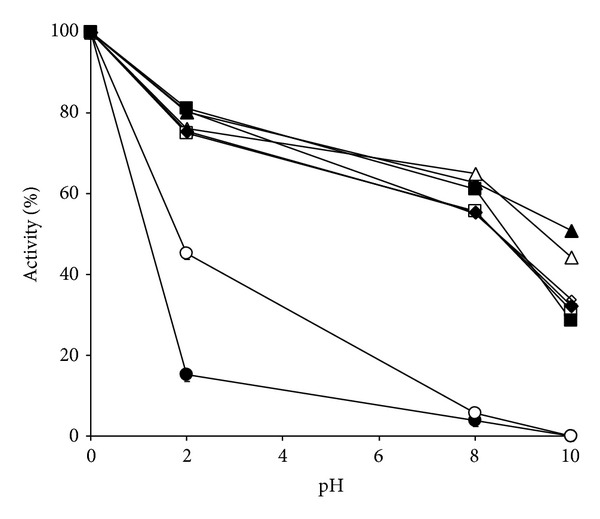
The effects of preincubation pHs of 4 (●), 5 (◯), 6 (▲), 7 (Δ), 7.5 (◊), 8 (□), 9 (■), and 10 (◆) on the stability of the Mo-reducing enzyme. The enzyme was preincubated at different pHs on ice before assay. Error bars are mean ± standard deviation of triplicates.

Figure 8 shows that the Mo-reducing enzyme was relatively unstable at all of the preincubated pH studied after 10 hours of incubation on ice with a lowering of activity of up to 50% at all incubation pHs. Acidic preincubation pH (pH 4 and 5) causes the most damage with total loss of activity after 10 hours. Mo-reducing enzyme is most stable at pH 6.0. This instability is probably the main reason why a large loss of activity was seen during chromatography where the temperature could reach as high as 10°C especially during transfer and handling. The composite effect is a reduction of activity not due to proteases of loss of cofactor but due to the instability of the enzyme itself. The most useful buffer at this pH for storage was citrate (pKa 5.0) at pH 6.0 although phosphate (pKa 6.8) can be used but only at lower molarities (<20 mM) since it is known to destabilize phosphomolybdate when present in the reaction mixture [21].

Enzymes, being amphoteric molecules, contain on their surface a large number of basic and acidic groups. The charges on these basic and acidic groups vary according to their dissociation constants that vary with the pH of the environment. Changes in pH will affect the distribution of charges on their exterior surfaces and hence the total net charge of the enzymes. This in turn affects the reactivity of the catalytic active groups. During assay, change in pH of the environment would change the ionic state of the catalytic groups especially in the active sites. This will render usually unfavorable binding to the substrate(s) in the extreme conditions. This is the cause of enzymes having a pH profile in accordance with the optimum and inactivation pH range. In the case of stability, as in this study, changes in charges could be permanent after prolonged storage in unfavorable pH or buffer species and will affect the structural stability and solubility of the enzyme, leading towards inactivity or denaturation of the enzyme.

### 3.6. Determination of Kinetic Parameter

Preliminary results show that 15 mM 12-MP were saturating. A plot of initial rates against substrate concentrations at 15 mM 12-MP registered a *V*_max⁡_ for NADH at 12.0 nmole Mo blue/min/mg protein. The model giving the best regression coefficient (0.99) was one phase binding. The apparent *K*_*m*_ for NADH was 0.79 mM.

At 5 mM NADH, the apparent *V*_max⁡_ and apparent *K*_*m*_ values for 12-MP of 12.05 nmole/min/mg protein and 3.87 mM, respectively, were obtained. The *V*_max⁡_ for NADH reported in this work is higher than EC 48's Mo-reducing enzyme at 6.28 nmole Mo blue/min/mg protein [19] while the apparent *K*_*m*_ for NADH was lower than EC 48 at 1.65 mM, suggesting a higher affinity to the Mo-reducing enzyme in strain Dr.Y5. A similar apparent *V*_max⁡_ values for the electron accepting substrate, 12-MP were obtained but a higher apparent *K*_*m*_ for this substrate compared to 0.32 mM for EC 48's Mo-reducing enzyme [19] suggests that the enzyme from EC 48 has a higher affinity to 12-MP compared to this strain. The catalytic efficiency (*k*_cat_/*K*_*m*_) of the Mo-reducing enzyme was 5.47 M^−1^ s^−1^. The catalytic efficiency is about 200,000-fold less efficient than the most catalytically efficient chromate reductase, a similarly related metal reductase, isolated from *Thermus scotoductus* [29]. The similarity and difference seen in this work reflect the unique nature of Mo-reducing enzyme from different bacteria.

### 3.7. Effects of Metal Ions and Stability Compounds

The addition of EDTA was inhibitory to the enzyme activity while the sulfhydryl protective agents such as DTT and 2-mercaptoethanol increase activity at 1 mM and were strongly inhibitory at 5 mM (Table 2). The effect of metal ions shows that both lead and copper inhibited the enzyme (Table 3). The Mo-reducing enzyme probably contains metals cofactor as evident from the effect of EDTA. The enzyme also probably contains the sulfhydryl group since the addition of sulfhydryl protective agents such as DTT and 2-mercaptoethanol increases activity. The inhibitory effect of copper on the Mo-reducing enzyme is also demonstrated in all recently isolated Mo-reducing bacteria [9–16] and in other heavy metals-reducing bacteria works such as chromium [30, 31] and mercury [32]. It is known that copper binds itself to the sulfhydryl group of enzymes [26] and this is probably the likely scenario in this enzyme.

## 4. Conclusion

In this work, the purification of the Mo-reducing activity is reported using a combination of ion exchange and gel filtration. Since its first report in *E. coli* in 1896, the Mo-reducing enzyme that is responsible for the molybdenum blue phenomenon has never been purified. However, work in this area is yet to be completed. The enzyme yield is such that it is not possible to do N-terminal sequencing. Thus, works are currently being carried out to maximize the yield. If the sequencing results showed novel characteristics, then the Mo-reducing enzyme should have a new name, NAD(P)H: phosphomolybdate oxidoreductase or, simply, phosphomolybdate reductase. The greater understanding of the underlying mechanism of molybdenum reduction catalyzed by this enzyme could lead to improved processes in the area of bioremediation [33], reducing biocorrosion [34], biomining [35], and biorecycling of molybdenum [36, 37].

## Figures and Tables

**Table 1:** Partial purification scheme of Mo-reducing enzyme from *Serratia *sp. strain DRY5.

Fraction	Total protein (mg)	Specific activity (units/mg protein)	Total activity (units)	Yield %	Fold purification
Crude	500	3	1500	100.0	1.0
Mono Q	120	11.5	1380	92	3.8
Mono Q*	10	17.5	175	11.66	5.8
Zorbax GF-250	1.2	24.3	29.16	1.9	8.1
Chromatofocusing	0.1	0.25	0.25	0.0002	0.08

**Table 2:** Effect of denaturants and stabilising agents on activity of Mo-reducing enzyme from *Serratia *sp. strain DRY5.

Compounds	Concentrations which decrease enzyme activity by 50%	Concentrations which increase enzyme activity by 50%
EDTA	0.1 mM	—
Triton x-100	0.1%	—
SDS	0.1%	—
Ethylene glycol	>10%	—
Ethanol	>10%	—
Acetone	>10%	—
DTT	10 mM	1 mM
2-Mercaptoethanol	5 mM	1 mM

**Table 3:** Effect of metal salts (0.01 mM) on activity of Mo-reducing enzyme of *Serratia *sp. strain DRY5.

Metals	Relative activity (%) ± standard deviation (*n* = 3)
Control	101.07 ± 2.10
Ni	103.86 ± 2.51
Ag	103.25 ± 5.19
Co	103.11 ± 2.83
Cd	102.30 ± 2.79
W	102.22 ± 6.28
Zn	101.61 ± 3.71
Al	101.61 ± 2.59
Cr	101.47 ± 3.96
Cs	101.32 ± 5.15
As	101.07 ± 6.57
Li	98.30 ± 4.89
Se	97.38 ± 2.30
Bo	96.89 ± 2.12
Hg	96.83 ± 2.55
Ba	93.84 ± 2.83
Mn	92.34 ± 10.77
Pb	61.93 ± 25.35
Cu	23.32 ± 1.55
